# CTHRC1: A New Candidate Biomarker for Improved Rheumatoid Arthritis Diagnosis

**DOI:** 10.3389/fimmu.2019.01353

**Published:** 2019-06-12

**Authors:** Askhat Myngbay, Yergali Bexeitov, Altynai Adilbayeva, Zhenisbek Assylbekov, Bogdan P. Yevstratenko, Rysgul M. Aitzhanova, Bakhyt Matkarimov, Vyacheslav A. Adarichev, Jeannette Kunz

**Affiliations:** ^1^PhD Program in Science, Engineering and Technology, Nazarbayev University, Astana, Kazakhstan; ^2^National Laboratory Astana, Astana, Kazakhstan; ^3^National Laboratory Astana, Department of General Biology and Genomics, Faculty of Natural Sciences, L. N. Gumilyov Eurasian National University, Astana, Kazakhstan; ^4^Department of Biology, Nazarbayev University, Astana, Kazakhstan; ^5^Department of Mathematics, Nazarbayev University, Astana, Kazakhstan; ^6^Republican Diagnostic Center, Astana, Kazakhstan; ^7^Department of Medicine (Division of Rheumatology), Department of Microbiology & Immunology, Albert Einstein College of Medicine, Bronx, NY, United States

**Keywords:** rheumatoid arthritis, biomarker, collagen triple helix repeat containing 1, (CTHRC1), rheumatoid factor (RF), anti-citrullinated protein antibodies (ACPA), disease activity score DAS28

## Abstract

**Background:** The purpose of this study was to determine whether plasma levels of the collagen triple helix repeat containing 1 (CTHRC1) protein can serve as a blood-based biomarker for improved diagnosis of rheumatoid arthritis (RA) patients and monitoring of RA disease activity.

**Methods:** We measured levels of CTHRC1 in the plasma of patients diagnosed with RA, osteoarthritis (OA), reactive arthritis (ReA), as well as in healthy individuals. We then assessed the correlation between CTHRC1 protein and a range of indices including the 28-joint disease activity score (DAS28), rheumatoid factor (RF), C-reactive protein (CRP), anti-citrullinated protein antibodies (ACPA), erythrocyte sedimentation rate (ESR), as well as a panel of cytokines, including interleukin 1 beta (IL-1β), interleukin 6 (IL-6), interleukin 8 (IL-8), and interferon gamma (IFNγ). Receiver operating characteristic (ROC) analysis was further performed to assess the diagnostic value of CTHRC1.

**Results:** CTHRC1 plasma levels were significantly elevated in RA patients compared to healthy individuals, OA and ReA patients. ROC curve and risk score analysis suggested that plasma CTHRC1 can accurately discriminate patients with RA from healthy controls and may have practical value for RA diagnosis. CTHRC1 levels were positively associated with RF, ACPA, CRP, and disease activity based on the combined index of DAS28 with CRP (DAS28-CRP), and also strongly correlated with IL-1β, IL-6, IL-8, and IFNγ.

**Conclusion:** Our studies show that CTHRC1 is a sensitive and easy-to-measure plasma marker that differentiates between RA and healthy status and also distinguishes between RA and other forms of arthritis, such as OA and ReA. At the current level of understanding, plasma CTHRC1 levels may improve the diagnosis of RA and these findings warrant confirmation in a larger, more comprehensive patient population.

## Background

Rheumatoid arthritis (RA) is a chronic, progressive, autoimmune disease of synovial joints. Disease progression is characterized by periods of flares with high disease activity involving both, a systemic immune response and tissue-specific inflammatory events that can lead to erosive joint and bone destruction and subsequent disability (1, 2). Mounting evidence indicates that early RA diagnosis and treatment aimed at controlling disease activity is essential in halting or delaying progression to erosive disease (1, 3). This so-called “treat-to-target” strategy relies on accurate diagnosis of patients early in the disease course and frequent monitoring of disease activity thereafter in order to guide and optimize treatment to achieve remission or a low disease activity state (1, 3).

However, early diagnosis of RA and clinical assessment of disease activity in individual patients remains a challenge. RA is a multifactorial disease with significant contribution from genetic and non-genetic factors (4–7) that altogether account for a complex disease pathology. There is considerable patient-to-patient variability in clinical presentation of RA, for example in the number of affected joints, the presence or absence of specific serological markers, and the extent of joint and bone degradation (1, 8, 9). Disease heterogeneity is further evident in the incomplete treatment responses observed in many patients (5, 10). Indeed, there is mounting evidence that the heterogeneity of RA reflects genetic and biological differences and that multiple molecularly distinct RA subtypes may exist, which differ in their underlying disease mechanisms (11–13).

Despite the increasing appreciation of disease heterogeneity, there is a lack of biomarkers for stratification of RA patients in clinical practice. Currently, diagnosis of RA is based mainly on the presence and high titers of rheumatoid factor (RF) and of antibodies against citrullinated protein (ACPA) or against cyclic citrullinated peptide (anti-CCP) in the plasma (14). Other classification criteria for RA are based on the extent of tenderness and swelling of the joints and levels of acute-phase reactants such as CRP and ESR. The major RF species, IgM and IgA RFs, are detected in 60–70% of RA patients (15). Patients with high RF typically have higher disease activity and develop more aggressive forms of the disease with greater disability (14). However, RF is often absent in early stages of the disease and is not a specific marker for RA, but can also be found in other autoimmune and inflammatory diseases (14). ACPA and CCP autoantibodies on the other hand provide high specificity for RA (90–95%), but moderate sensitivity (60–75%) (14, 16, 17). In addition, CRP and ESR are not specific for RA, but are general indicators of inflammation that can be elevated due to a variety of causes including age and systemic inflammatory activity (3, 4). Consequently, the limited number of biomarkers available often cannot differentiate patients from healthy subjects with a high degree of specificity early on in the disease course and may not accurately measure disease activity.

The search for improved RA biomarkers resulted in the identification of panels of prognostic markers, such as the Multi-Biomarker Disease Activity (MBDA) test, which measures a panel of 12 plasma proteins (18, 19). However, the clinical value of this multi-biomarker test remains unclear. Thus far, clinical studies in thousands of RA patients have not shown consistent correlation of the MBDA score with clinically important measures, such as the DAS28-CRP score, and have failed to correctly predict structural joint damage and radiographic progression (18, 19). These results underscore the need for additional biomarkers or combinations thereof, perhaps selected for specific patient populations, to better reflect the biological and pathological heterogeneity of RA.

A key contributor to the pathogenesis of RA leading to bone damage is synovial hyperplasia. Persistent inflammation of the synovial joint tissue leads to the formation of new pathological tissue, termed a pannus, which invades and erodes adjoining cartilage and bone (20). Pannus formation constitutes the primary reason for edema, pain, tissue destruction, joint deformities and bone erosion in RA patients (20). Arthritic pannus is a multicellular vascularized tissue composed of cells of both mesenchymal and hematopoietic origin, including synovial fibroblasts, osteoclasts, endothelial cells, dendritic cells, monocytes/macrophages, as well as T and B cells, that contribute to the development and progression of joint and cartilage erosion through secretion of pro-inflammatory cytokines and tissue-degrading proteases. Fibroblast-like synoviocytes (FLS), particularly the invasive and migratory cadherin-11-positive subtype (21), are major components of synovial pannus tissue and are considered active drivers in the pathogenesis of RA (20, 22, 23). Proteins implicated in the pathophysiology of the synovium may, thus, represent one class of candidate biomarkers.

We recently reported that increased levels of collagen triple helix repeat containing 1 protein (CTHRC1) are strongly associated with the severity of murine proteoglycan-induced arthritis and collagen antibody-induced murine arthritis (CAIA) (24–27). We further showed that CTHRC1 expression in murine experimental arthritis is increased in the synovium and specifically detected in activated murine and human RA-FLS (25) located at the synovial intimal lining and at the bone-pannus interface (28). Of interest, CTHRC1 is a secreted modulator of Wnt signaling, which is a key regulator of joint remodeling (29), and promotes cell proliferation and migration (28). Therefore, CTHRC1 may contribute to multiple aspects of the pathogenic FLS behavior in RA and modulate processes that promote synovial hyperplasia and invasion. The expression pattern of CTHRC1 in pannus, its role in the function of FLS relevant to cartilage damage in RA, and CTHRC1's association with disease severity in murine arthritis raised the question of whether CTHRC1 could be used as a marker for RA diagnosis and monitoring of disease activity in patients.

To begin to address this question, we performed a cross-sectional pilot study to analyze the levels of CTHRC1 in the peripheral blood of patients diagnosed with RA, OA, and ReA, as well as in healthy controls, and to test for association of CTHRC1 with RA status and disease activity.

## Materials and Methods

### Study Population, Ethics Approval, and Consent to Participate

Fifty seven RA, sixty five OA, twelve ReA patients, and fourteen healthy controls were recruited from outpatients at the Republican Diagnostics Center, Astana, Kazakhstan. Blood collection for studies was performed as part of the diagnostic process during the clinical appointment. Demographical (age and sex) and disease-related parameters were recorded in all patients at the time of blood sampling. With respect to disease-specific parameters, disease duration, tender joint count 68, swollen joint count 66, and a patient global assessment were evaluated. In addition, CRP level, ESR and the presence of RF as well as ACPA were measured. Inclusion criteria: All RA patients fulfilled the 2010 ACR/EULAR classification criteria for RA (30), and their ages ranged from 30 to 75 years. The RA and OA patients underwent a clinical evaluation performed by a single rheumatologist. Diagnosis of RA patients was based on medical history, CBC, biochemical parameters, physical assessment of patient's tender and swollen joints and MRI scans of joints. Disease activity of RA patients was evaluated with the composite DAS28-CRP score and was calculated at the time of blood donation during the clinical appointment. Anti-rheumatic therapy and any other concomitant treatment were recorded at the same time. OA patients were included based on clinical diagnosis of primary OA of the knee(s) according to the physician's clinical judgment and the absence of other forms of arthritis. ReA patients had a diagnosis of acute arthritis affecting knees, ankles, or the heels just after suffering an infection and were negative for diagnostic parameters consistent with either RA or OA. Healthy individuals constituted outpatients who visited the clinic for routine health tests and were without prior history of chronic inflammation or any form of arthritis as evidenced by normal levels of CRP, ESR, undetectable levels of RF and ACPA, and normal knee MRIs. The analysis of clinical parameters including assessment of RF, ACPA, CRP, and ESR was performed in a blinded fashion by the certified central clinical laboratory at the Republican Diagnostic Center in Astana, Kazakhstan. The patient data was subsequently pseudonymized. The study was approved by the institutional review board at the Republican Diagnostic Center in Astana, Kazakhstan, and the Institutional Research Ethics Committee at Nazarbayev University, Astana, Kazakhstan (Protocol #N32) and complied with the International Ethical Guidelines for Biomedical Research Involving Human Subjects, Good Clinical Practice guidelines, the Declaration of Helsinki, and local laws. All patients provided written informed consent for the study and publication of the resulting data.

### CTHRC1 Immunodetection

Venous blood was collected into heparinized tubes, cells were removed by centrifugation at 1,000 × g for 10 min, plasma was stored at −70°C. A commercially available sandwich enzyme-linked immunosorbent assay (ELISA) for CTHRC1 quantification was performed in duplicates according to manufacturer protocol (www.mmcri.org/antibody, Maine Medical Center Research Institute, Scarborough, ME), as described previously (28). Briefly, 96-well Maxisorp plates (Nunc) were coated overnight at 4°C with 1.8 μg/ml capture antibodies 13E09 in a carbonate-bicarbonate buffer of pH 9.4. The next day, wells were washed with PBS-TB buffer (1 × PBS, 0.05% Tween 20, 0.1% BSA) and then blocked with the same buffer for 1 h. Human plasma rather than serum was assayed based on previous evaluation of the CTHRC1 ELISA assay showing superior sensitivity (28). Human plasma was diluted 1:5 to 1:100 in PBS-TB and incubated with absorbed capture antibodies for 2 h. Subsequently, the wells were washed and then incubated with the biotinylated detection antibody Vli10G07 diluted 1:500 in PBS-TB for 1 h. After washing, wells were incubated for 1 h with streptavidin conjugated with horseradish peroxidase (Thermo Fisher Scientific) diluted at 1:8,000 in PBS-TB. After a final wash, TMB 3,3′,5,5′- tetramethylbenzidine chromogenic substrate (Amresco) was added, and absorbance was measured at 450 nm. Absorbance was converted to absolute concentration using recombinant CTHRC1 protein (rhCTHRC1) provided in the kit as a reference. Sensitivity of the ELISA is better than 1 ng/ml level according to manufacturer's protocol.

### Evaluation of CTHRC1 Protein Stability

To study CTHRC1 proteolysis, human plasma was spiked with rhCTHRC1 (5 ng/μL rhCTHRC1, 12% plasma or synovial fluid (SF), 1 x PBS) and incubated at 37°C. Samples were denatured at 97°C in a Laemmli sample buffer and separated on 12% SDS-PAGE. Polyvinylidene fluoride membranes (Merck Millipore) with transferred protein were blocked in 5% (w/v) dry milk for 1 h in PBST and then probed with rabbit antibodies to CTHRC1 (Vli55, www.mmcri.org/antibody) and were developed with secondary HRP–conjugated goat anti-rabbit (Sigma) antibodies. Enhanced chemiluminescent substrate (Thermo Fisher Scientific) and the BioSpectrum 800 Imaging System (UVP) were used to detect signal.

### Legendplex™ Multiplex Analyte Microsphere-Based Immunoassay

LEGENDplex™ Multianalyte immunoassay for IL-1b, IL-6, IL-8, IFNγ, and SCF was performed in accordance with manufacturer's instructions (LEGENDplex™, BioLegend). In brief, human plasma samples were centrifuged to remove debris and diluted 1:5 to 1:100 in PBS-TB and added to wells containing beads conjugated with analyte-specific antibodies. Detection antibodies were subsequently added to each well. After incubation of the plate for 2 h at room temperature with shaking, streptavidin-phycoerythrin was added, and plates were shaken for an additional 30 min. Finally, beads were washed twice with PBS-T using centrifugation at 1,000 g for 5 min to collect beads after each washing step. Standard solutions containing eight different concentrations of analytes (from 0 to 50,000 pg/mL) were used on each plate for standard curve determination and were incubated the same way as assay samples. After incubation and washing, beads were analyzed using BD FACSAria SORP flow cytometer (BD Biosciences). PMT voltages for Allophycocyanin (APC) and Phycoerythrin (PE) channels were set up immediately before the analysis using the Setup Beads provided in the kit according to manufacturer's instructions. Data analysis and calculations of concentration for samples based on the obtained standard curves was performed using LEGENDplex™ data analysis software according to manufacturer's instructions.

### Statistical Analysis

Patient data are summarized as the mean with standard deviation (SD) or as medians and interquartile ranges (IQR). Comparisons of patient group and gender was done with an ANOVA and *t*-test if the distribution was normal, if not with the Kruskal Wallis test and Mann-Whitney *U*-test, respectively. Pearson's Chi-square test was performed for qualitative variables. The Kruskal-Wallis with Dunn's *post-hoc* test with or without Bonferroni correction was used to determine differences across groups for CTHRC1. Non-parametric Spearman correlation coefficients were used to describe the association between two continuous variables. ROC Curves analysis and AUC estimation were also performed in order to determine the best threshold, which discriminates our group of interest thanks to the Youden index. Linear logistic regression analysis was performed to evaluate the association between plasma CTHRC1 levels and RA in terms of unadjusted odds ratio (OR). CTHRC1 levels followed a skewed distribution and were log transformed when used as continuous variables. Data were >95% complete. All reported *P*-values were two-tailed, with *P* ≤ 0.05 being considered significant. Statistical analyses and graphic illustrations were performed under GraphPad Prism version 6.03 for Windows (GraphPad Software, La Jolla California USA) and R (v3.5.1), using ggplot2 and pROC libraries.

## Results

### Patient Demographics and Clinical Characteristics

Fifty seven RA outpatients of the Republican Diagnostics Center, Astana, Kazakhstan, were recruited for this study. All RA patients fulfilled the 2010 ACR/EULAR classification criteria for RA (30). As reference populations, we used 65 patients with OA, 12 patients with ReA, and 14 healthy individuals. The average age of subjects in the RA population was 49.5 years with an average age of RA onset at 42.0 years (Table 1). To comply with female preponderance in RA, the study enrolled mostly female patients (89.5%, Table 2). The majority of RA patients in the studied cohort (54.25%) were undergoing treatment with methotrexate or metoject (Mtx, Table 2) at the time of blood and data collection. Other subgroups of RA patients were treated with methotrexate in combination with glucocorticoids (MTx+GC, 17.5%, Table 2), methotrexate in combination with other synthetic DMARDs (MTx+DMARD, 5.25%, Table 2), or methotrexate with other treatments (NSAIDs plus synthetic DMARDs, with or without ibandronate; MTx+, 3.5%, Table 2). 3.5% of patients received glucocorticoids alone (prednisolone or methylprednisolone, GC, Table 2), whereas leflunomide or hydroxychloroquine alone or in combination were prescribed for an additional 3.5% of patients (DMARD, Table 2). 1.75% of patients were treated with a combination of methotrexate and non-steroidal anti-inflammatory drugs (MTX+NSAIDs, Table 2). The remainder of patients (1.75%) received sulfasalazine (Ssz, Table 2). No patients were treated with biologics. Nine percentage of patients had not yet received any treatment at the time of blood and data collection.

**Table 1:** Patient demographic and clinical characteristics.

Parameter^*^	**RA (*n* = 57)**	**OA (*n* = 65)**	**ReA (*n* = 12)**	**Healthy (*n* = 14)**	**RA-OA**	**RA-ReA**	**RA-He**
Age (years)	49.51 ± 13.65	56.78 ± 1 0.13	37.33 ± 9.58	34.5 ± 10.99	0.009	0.016	0.002^@^
Sex							
F	51 (89.47%)	56 (86.15%)	10 (62.50%)	12 (85.71%)			
M	6 (10.53%)	9 (13.85%)	6 (37.50%)	2 (14.29%)			
COMPLETE BLOOD COUNT WITH DIFFERENTIAL^*^							
Leukocytes (mil/mL)	6.7 ± 2.41	6.31 ± 1.37	5.59 ± 1.3	6.46 ± 1.2	>0.999	0.283	>0.999^@^
Hemoglobin (g/L)	124.49 ± 14.31	133.8 ± 12.67	139.8 ± 18.24	135.64 ± 7.47	<0.001	0.004	0.012^@^
Erythrocytes (mil/μL)	4.45 ± 0.48	4.63 ± 0.53	4.89 ± 0.45	4.5 ± 0.35	0.12	0.005	>0.999^@^
Platelets (mil/mL)	292.51 ± 85.31	253.2 ± 64.97	252.5 ± 63.84	260 ± 31.39	0.006	0.241	0.906^@^
Basophils (mil/mL)	0.04 ± 0.02	0.04 ± 0.02	0.04 ± 0.026	0.04 ± 0.02	0.603	>0.999	<0.001^#^
Lymphocytes (mil/mL)	1.79 ± 0.69	2.04 ± 0.53	1.98 ± 0.5	2.01 ± 0.32	0.071	0.896	<0.001^@^
Monocytes (mil/mL)	0.54 ± 0.23	0.49 ± 0.15	0.47 ± 0.18	0.46 ± 0.12	0.912	>0.999	<0.001^@^
Neutrophils (mil/mL)	4.11 ± 1.92	3.53 ± 0.98	2.8 ± 0.73	3.78 ± 0.94	0.627	0.026	>0.999^@^
Eosinophils (mil/mL)	0.15 ± 0.19	0.19 ± 0.11	0.20 ± 0.12	0.14 ± 0.08	<0.001	0.029	0.874^#^
BLOOD ASSAYS^*^							
CRP (mg/L)	14.72 ± 17.45	4.9 ± 11.19	1.87 ± 1.6	0.83 ± 0.85	0.0003	0.001	<0.001^#^
ESR (mm/h)	20.6 ± 12.47	16.46 ± 9.56	8.58 ± 4.1	6.31 ± 2.14	0.483	0.001	<0.001^@^
RF (u/ml)	62.88 ± 66.11	10.42 ± 8.19	7.69 ± 3.9	n.a.	<0.001	<0.001	n.a.
ACPA (u/ml)	135.56 ± 212.33	0.38 ± 0.53	0.26 ± 0.4	n.a.	<0.001	<0.001	n.a.
CTHRC1 (ng/ml)							
Mean	20.39 ± 25.38	1.78 ± 4.49	2.15 ± 2.54	2.29 ± 3.73	<0.001	<0.001	<0.001^#^
Median [Q1–Q3]	8.28 (0–96)	0 (0–20.55)	3.27 (0–17.68)	0.05 (0–11.0)	<0.001	<0.001	<0.001^#^
CLINIC^*^							
DAS28-CRP	3.79 ± 0.85						

* Average values with standard deviation (±SD) are presented. p-values for statistical difference between indicated parameters were calculated using

@ ANOVA or

# *Kruskal-Wallis testing. n.a., —parameter not determined. ESR, erythrocyte sedimentation rate; CRP, C-reactive protein; RF, rheumatoid factor; ACPA, anti-citrullinated protein antibodies; DAS28-CRP, disease activity score based on CRP*.

**Table 2:** RA Patient demographic and clinical characteristics.

	**Total**	**Men**	**Women**
**COMPLETE BLOOD COUNT**			
Leukocytes (mil/mL)	6.7 ± 2.4	9.27 ± 1.6	6.41 ± 2.28 (^**^)
Hemoglobin (g/L)	124.5 ± 14.31	136.5 ± 10.9	123.07 ± 13.84 (^*^)
Erythrocytes (mil/μL)	4.45 ± 0.47	4.77 ± 0.29	4.41 ± 0.47 (^*^)
Platelets (mil/mL)	292.5 ± 85.31	307.3 ± 96.48	290.76 ± 82.86
Basophils (mil/mL)	0.04 ± 0.024	0.05 ± 0.02	0.037 ± 0.03
Lymphocytes (mil/mL)	1.79 ± 0.69	2.22 ± 0.45	1.74 ± 0.68
Monocytes (mil/mL)	0.54 ± 0.23	0.85 ± 0.25	0.5 ± 0.19 (^*^)
Neutrophils (mil/mL)	4.1 ± 1.9	5.8 ± 1.18	3.91 ± 1.87 (^*^)
Eosinophils (mil/mL)	0.15 ± 0.19	0.46 ± 0.38	0.11 ± 0.09
**BLOOD ASSAYS**			
ESR (mm/h)	20.6 ± 12.47	18.83 ± 12.02	20.8 ± 12.38
CRP (mg/L)	14.72 ± 17.45	29.81 ± 19.07	12.94 ± 16.17
RF (U/ml)	62.88 ± 66.11	62.54 ± 50.19	62.91 ± 67.09
ACPA (U/ml)	135.6 ± 212.3	114.82 ± 118.5	138.01 ± 218.6
CTHRC1, ng/ml	20.39 ± 25.38	33.36 ± 31.72	18.86 ± 23.8
**CLINIC**			
*N*	57	6	51
Age	49.51 ± 13.65	47.83 ± 15.62	49.71 ± 13.24
DAS28-CRP	3.78 ± 0.85	3.94 ± 0.58	3.76 ± 0.87
Age of onset (years)	42.0 ± 14.58	36.66 ± 13.8	42.63 ± 14.54
Duration of disease (years)	8.04 ± 8.18	11.5 ± 10.07	7.67 ± 7.8
Duration of treatment (years)	5.05 ± 6.27	8.76 ± 9.39	4.41 ± 5.3
Tender joint count (0-28)	8.12 ± 3.07	8.16 ± 3.28	8.10 ± 3.04
Swollen joint count (0-28)	2.12 ± 2.27	1.16 ± 0.68	2.24 ± 2.38 (^*^)
**TREATMENT**			
Mtx	54.25%	n.a.	n.a.
MTX + GC	17.5%	n.a	n.a
Mtx + DMARDs	5.25%	n.a.	n.a.
Mtx + NSAIDs	1.75%	n.a	n.a
GC	3.5%	n.a.	n.a.
DMARDs	3.5%	n.a	n.a
Mtx+	3.5%	n.a.	n.a.
SSZ	1.75%	n.a	n.a
None	9.0%	n.a	n.a

^*^p-values for statistical difference between men and women parameters were calculated using Mann–Whitney U-test:

* *p < 0.05*;

** *p < 0.01*.

*Average values with standard deviation (±SD) are presented. n.a. —parameter not determined. ESR, erythrocyte sedimentation rate; CRP, C-reactive protein; RF, rheumatoid factor; ACPA, anti-citrullinated protein antibodies; DAS28-CRP, disease activity score based on CRP. Mtx, methotrexate or metoject; Mtx + GC, methotrexate in combination with glucocorticoids; Mtx + DMARDs, methotrexate in combination with other synthetic DMARDs (leflunomide or hydroxychloroquine); Mtx + NSAIDs, methotrexate in combination with non-steroidal anti-inflammatory drugs; Mtx +, methotrexate in combination with NSAIDs and synthetic DMARDs, with or without ibandronate; GC, glucocorticoids (prednisolone or methylprednisolone); DMARDs, leflunomide or hydroxychloroquine alone or in combination; SSZ, sulfasalazine*.

RF and ACPA were significantly increased in autoimmune inflammatory RA vs. OA or ReA (approximately 6-fold (RF) and >350-fold (ACPA), *p* < 0.001), whereas CRP levels were approximately 3-fold elevated in the RA vs. OA patient group and 8-fold higher in the RA vs. ReA group (Table 1). Among the RA population, women and men exhibited similar clinical parameters: DAS28-CRP was 3.94 in men vs. 3.76 in women and patients' age, CRP, RF and ACPA were similar (Table 2). Despite the treatment, an average DAS28-CRP score of 3.78 indicates that RA patients still exhibited moderate disease activity (Table 1). Hemoglobin, white blood cells, erythrocytes, and absolute numbers of monocytes (MON) and neutrophils (Ne) were up to 1.5-fold lower in females compared to males (*p* < 0.05, Table 2).

### CTHRC1 Plasma Levels Are Significantly Elevated in RA Patients

**Figure 1:**
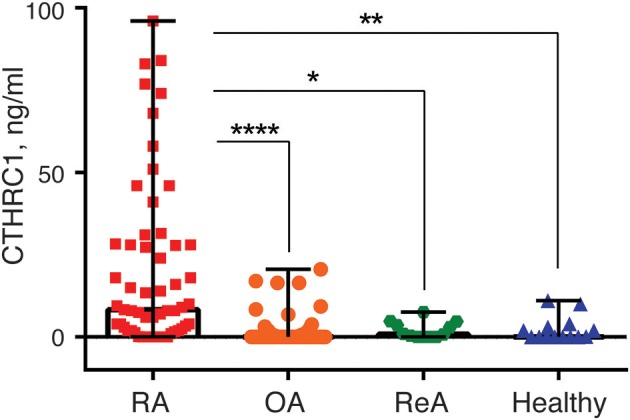
Blood plasma CTHRC1 levels in RA-patients and non-RA control groups. Plasma CTHRC1 concentration was measured using sandwich ELISA and recombinant CTHRC1 protein as a reference. Kruskal-Wallis with Dunn's *post-hoc* testing revealed a high statistical significance for the difference between RA (red squares) and healthy individuals' plasma (blue diamonds), as well as between RA and OA (orange circles), or ReA (green octagons) patients' plasma. Each diamond, octagon, square or circle corresponds to one patient. Box-and-whisker plot shows the median CTHRC1 levels within interquartile range and Tukey fences at 1.5 × IQR. The corresponding *p*-values are presented with asterisks: ^*^p < 0.05, ^**^p < 0.01, ^****^p < 0.0001; Kruskal-Wallis with Dunn's *post-hoc* test and Bonferroni adjustment.

To study circulatory levels of CTHRC1, we collected peripheral blood samples from RA, OA, and ReA patients, as well as from healthy individuals, and performed sandwich ELISA to measure plasma CTHRC1. We then compared CTHRC1 levels across patient groups and healthy controls. We found that, in the absence of detectable inflammatory disease, CTHRC1 is present only at low levels in the circulation: in the healthy control population, detectable levels of CTHRC1 reached an average of 2.29 ng/ml and a median level of 0.05 ng/ml (Kruskal-Wallis test, *p* < 0.001, Figure 1). These values are in agreement with a published report on CTHRC1 levels in thousands of healthy individuals, which showed that approximately one third of assayed plasma samples were negative for CTHRC1, whereas basal CTHRC1 levels in the remaining samples were low (31). OA and ReA patients exhibited a similar range of circulating CTHRC1 protein as the healthy control group. Accordingly, OA patients had an average plasma CTHRC1 level of 1.78 ng/ml and a median level of 0 ng/ml (Kruskal-Wallis test, *p* < 0.001, Figure 1), whereas ReA patients had an average level of 2.15 ng/ml and a median level of 3.27 ng/ml, respectively (Kruskal-Wallis test, *p* < 0.001, Figure 1). In stark contrast, circulating CTHRC1 levels were significantly increased in the RA cohort when compared to either control, OA or ReA groups with an average level of 20.39 ng/ml, a median level of 8.28 ng/ml, and a maximum level of 96 ng/ml (Kruskal-Wallis test, *p* < 0.001, Figure 1). There was no statistically significant difference in CTHRC1 levels between the Mtx and Mtx+GC treatment groups of the RA cohort (data not shown). When median CTHRC1 plasma levels were compared across cohorts, CTHRC1 levels were significantly elevated in RA patients compared to healthy controls (Dunn's test, Bonferroni adjusted *p* < 0.001, Figure 1) and OA patients (Dunn's test, Bonferroni adjusted *p* < 0.001, Figure 1). Elevated plasma CTHRC1 levels could further distinguish RA patients from ReA, (Dunn's test, *p* = 0.023), however, this trend was not confirmed by Bonferroni adjustment (adjusted *p* = 0.068, Figure 1). Overall, these data suggest that CTHRC1 levels are specifically and markedly elevated in RA compared to OA and healthy individuals.

Considering that CTHRC1 is produced by and secreted from stromal cells of different origin including stromal cells of pannus (28), we compared levels of CTHRC1 in plasma and synovial fluids collected from five RA patients. We found that in four out of the five patients the concentration of CTHRC1 in synovial fluid was similar or greater to the plasma concentration (Table 1S), which may indicate that pannus tissue is an important source of circulating CTHRC1.

### Evaluation of CTHRC1 Protein Stability

**Figure 2:**
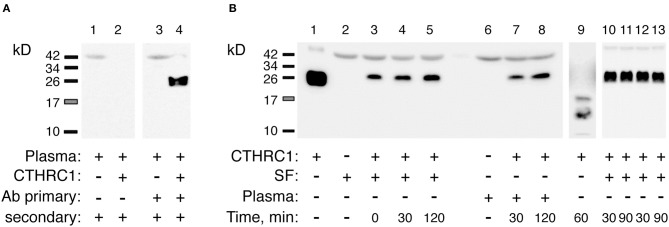
Detection and stability of plasma CTHRC1. **(A)** The specificity of the immunodetection was tested using 25 ng rhCTHRC1 spiked into the plasma (3 μL) of RA patients. Protein ladder bands (kD) are shown. **(B)** The resistance of the protein to proteolysis was tested with rhCTHRC1 spiked into synovial fluid (SF) or the plasma of RA patients. Final concentration of SF or plasma in the test was 12%, final amount of rhCTHRC1 loaded per lane was 15 ng. Lane 1 shows 50 ng of recombinant rhCTHRC1 protein as a reference. The Vli55 antibody was used for CTHRC1 immunodetection followed by appropriate secondary antibodies. As a positive control for digestion, incubation with trypsin was performed (lane 9). Lanes 10, 11: SF was heated for 30 min at 65°C (time, min) and then incubated with rhCTHRC1. Lanes 12, 13: SF was pre-heated for 30 min at 37°C.

To rule out the possibility that circulatory CTHRC1 protein in RA patients has a different half-life compared to healthy controls and OA patients, for example due to differences in protease content in the blood, we spiked recombinant human CTHRC1 (rhCTHRC1) into samples of plasma or synovial fluid, incubated the samples at 37°C for up to 2 h, and tested protein integrity using SDS-PAGE and immunodetection (Figure 2A). The incubation of rhCTHRC1 with plasma or synovial fluid from RA patients showed no degradation of the protein (Figure 2B). Likewise, no significant degradation of CTHRC1 was observed when rhCTHRC1 was incubated with plasma from OA patients or healthy controls indicating that the low levels of CTHRC1 detected in the plasma of OA patients and healthy controls is not due to an increased degradation rate (data not shown). We conclude that under the chosen assay conditions, CTHRC1 is stable in circulation and that ELISA assays can accurately measure levels of CTHRC1 in either the blood or synovial fluid.

### CTHRC1 Diagnostic Value for RA Identification

**Figure 3:**
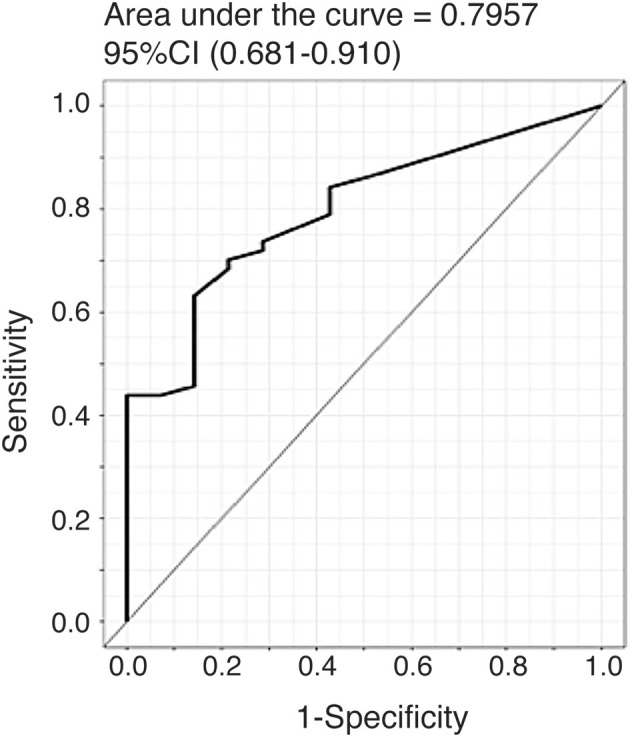
ROC curve for the risk prediction model. Receiver operating characteristic (ROC) curve analysis to assess the association of plasma CTHRC1 levels with RA vs. healthy status. The area under the curve was 0.796 for log(CTHRC1) (*p* = 0.032). Plots indicate individual protein abundances in patients.

To further evaluate the ability of plasma CTHRC1 to distinguish patients and healthy controls, we performed ROC curve analysis. The area under the ROC curve (AUC) was 0.796 (95% CI: 0.681–0.910) for CTHRC1 (Figure 3) based on binary logistic regression of log(CTHRC1) RA vs. healthy, indicating an acceptable discriminating power of the model. The optimal cut-off point for CTHRC1 to differentiate RA from healthy was 5 ng/ml based on the Youden's index reflecting a sensitivity of 62% and a specificity of 86% (accuracy = 68%, Table 3). The positive predictive value was 0.95 (95% CI: 0.88–1.00), whereas the negative predictive value was 0.36 (95% CI: 0.20–0.53, Table 3). Calculation of the odds ratio (OR) showed that patients testing positive on CTHRC1 have a 1.38-fold increased risk for a one unit increase in log(CTHRC1), which corresponds to an increase of ~2.7 ng/ml on the original scale (OR = 1.38, 95%CI: 1.14–1.68, *p* = 0.0011, Table 4). Overall, these results suggest that plasma CTHRC1 is a sensitive marker that may have practical value for RA diagnosis.

**Table 3:** Receiver operating characteristic (ROC) analysis of CTHRC1 in RA.

**ROC curve**	
AUC	0.796
AUC 95% CI	0.681–0.910
Cutoff	5 ng/ml
*P*-value	0.004
Sensitivity, %	62
Specificity, %	86
Accuracy	68
PPV, %	95
NPV, %	36

*AUC, area under the curve; CI, confidence interval; NPV, negative predictive value; PPV, positive predictive value; ROC, receiver operating characteristic*.

**Table 4:** Binary logistic regression results of CTHRC1 plasma levels for rheumatoid arthritis diagnosis.

**Parameter**	**β**	**S.E**.	**Wald**	**OR**	**OR 95% CI**	***P*-value**
log(CTHRC1)	0.324	0.099	10.693	1.382	1.138-1.678	0.001

*β, coefficient of logistic regression; S.E., standard error of β value; Wald, Wald value of Wald tests; OR, odds ratio; OR 95% CI, 95% confidence interval of OR value*.

### CTHRC1 Levels Are Associated With RF and ACPA and May Correlate With Disease Activity

**Figure 4:**
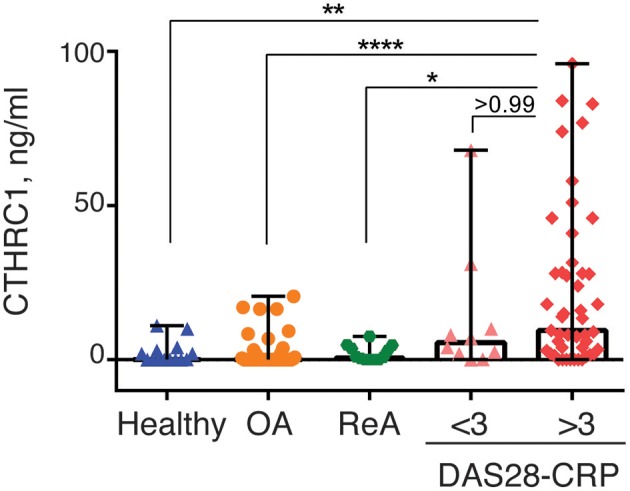
CTHRC1 plasma levels correlate with the arthritis severity score. CTHRC1 levels were calculated separately for healthy control (green circles) and OA (blue squares) groups, as well as for RA patients with a DAS28-CRP score <3 (<3, orange triangles), and >3 (>3, red diamonds). Each colored circle, square, triangle, or diamond corresponds to one patient in each group. Box-and-whisker plot shows the median CTHRC1 levels within interquartile range and Tukey fences at 1.5 × IQR. Statistically significant differences between RA groups vs. healthy controls and the OA patient cohort are indicated with asterisks: ^**^p < 0.01, ^****^p < 0.0001, Kruskal-Wallis with Dunn's *post-hoc* test and Bonferroni adjustment; ^*^p < 0.05, Kruskal-Wallis test.

RA disease activity is categorized as being in remission (DAS28-CRP < 2.6), weak (DAS28-CRP < 3.2), moderate (DAS28-CRP > 3.2), or high (DAS28-CRP > 5) based on specific DAS28-CRP cut-off scores. Because the vast majority of RA patients in our study were undergoing treatment at the time of study and most patients were diagnosed with low to moderate disease activity, we divided patients with RA into two groups based on the DAS28-CRP score to evaluate the ability of CTHRC1 to monitor disease severity and activity: patients who were in remission or had low disease activity (DAS28-CRP < 3.0) and patients with moderate to high disease activity (DAS28-CRP > 3.0). As shown in Figure 4, these two groups differed in median plasma CTHRC1 levels: for DAS28-CRP < 3 CTHRC1 median (min-max) levels were 5.5 ng/ml (range: 0–68 ng/ml; *p* < 0.001), whereas for DAS28-CRP > 3, CTHRC1 median levels were 9.5 ng/ml (range: 0–96 ng/ml; *p* < 0.001). There was no statistically significant difference between the RA group with DAS28-CRP lower than 3 and the RA group with DAS28-CRP higher than 3 (*p* > 0.999).

When median plasma CTHRC1 levels were compared between the different groups, elevated plasma CTHRC1 levels were sufficient to differentiate RA patients with a DAS28-CRP > 3 from OA patients and from healthy controls (Bonferroni adjusted *p* < 0.001, Figure 4). The difference between the RA group with a DAS28-CRP > 3 and the ReA group was statistically significant after applying the *post-hoc* Dunn's test (*p* = 0.023), but was no longer significant after Bonferroni adjustment (adjusted *p* = 0.068, Figure 4).

**Figure 5:**
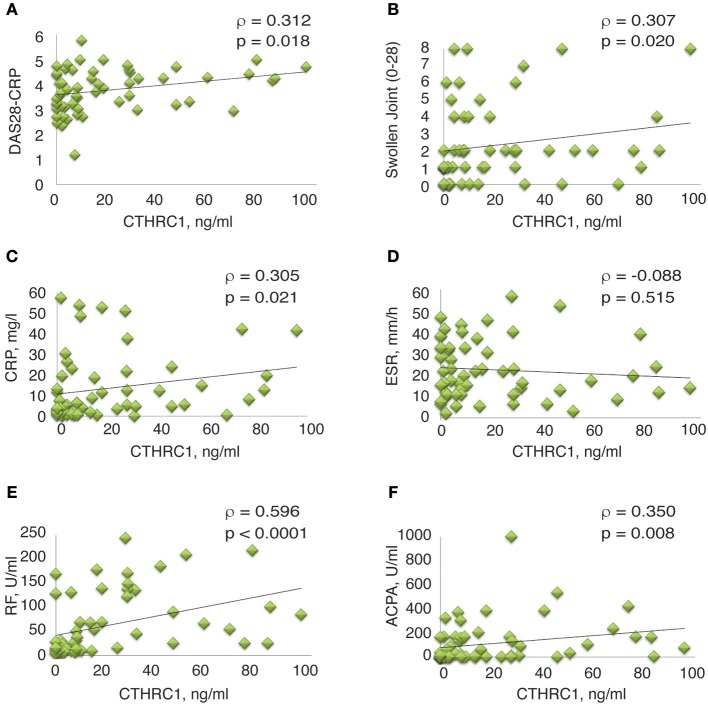
Correlation between clinical measures of RA and plasma levels of CTHRC1. **(A–E)** Correlation between plasma CTHRC1 and indicated clinical measures in the RA cohort. The y-axis reflects the plasma concentration of CTHRC1 in ng/ml. The x-axis reflects the DAS28-CRP score, the swollen joint (0–28) score, the concentration of RF and ACPA (both in U/ml), the concentration of CRP in mg/L, and the measurement of ESR in mm/hr. Scatter plot graphs showing correlation between **(A)** CTHRC1 and DAS28-CRP, **(B)** CTHRC1 and Swollen joint count (0–28), **(C)** CTHRC1 and CRP, **(D)** CTHRC1 and ESR, **(E)** CTHRC1 and RF, and **(F)** CTHRC1 and ACPA. Each RA patient's sample is represented with a green diamond. The relationship between variables was evaluated using the Spearman rank correlation test. Trend lines indicate linear correlation. Spearman's coefficient of the correlation (ρ) and the corresponding *P*-value are shown on each plot.

Spearman's rank correlation analysis confirmed the correlation between circulating CTHRC1 levels in RA patients and clinical DAS28-CRP score (ρ = 0.312, *p* = 0.018, Figure 5A and Table 2S). CTHRC1 levels significantly correlated with swollen joint count (ρ = 0.307, *p* = 0.02) and with CRP (ρ = 0.305, *p* = 0.021), but not with tender joint count (Figures 5B,C; Table 1S and data not shown). In addition, plasma CTHRC1 showed statistically significant positive correlation with RF (ρ = 0.596, *p* < 0.0001, Figure 5E and Table 1S) and ACPA (ρ = 0.35, *p* = 0.008, Figure 5F and Table 1S), but not with ESR, which exhibited a negative trend that did not reach statistical significance (ρ = −0.088, *p* = 0.512, Figure 5D; Table 1S). Overall, these findings show a statistically significant association between CTHRC1, RF, and ACPA. CTHRC1 also positively correlates with RA disease activity, however, this trend needs to be further investigated and validated using larger patient cohorts that cover the entire spectrum of RA disease activity.

### CTHRC1 Levels Correlate With Inflammatory Cytokines

To further investigate the link between circulatory CTHRC1 and disease status, we performed multi-analyte BioLegend immunoassays on a randomly chosen subset of twenty RA patients for levels of the inflammatory cytokines IL-1β, IL-6, IL-8, and IFNγ, which are increased in the plasma of patients with RA (6, 7) and are considered indicators of the inflammatory state. Even though methotrexate treatment is likely to influence the landscape of cytokine production in RA patients (32), we found a strong and statistically significant association between CTHRC1 and all four inflammatory cytokines tested (ρ = 0.59–0.91, *p* < 0.0001, Dunn's test; Table 5). IL-1β and IFNγ blood levels were also tightly correlated with each other (ρ = 0.88 and 0.91, respectively, *p* < 0.0001, Dunn's test; Table 5) and with IL-6, IL-8 (ρ = 0.59 and 0.86, respectively, *p* < 0.0001, Dunn's test; Table 5). Overall, these finding underscore the potential relevance of CTHRC1 as a marker in arthritis pathogenesis and also support our earlier findings showing positive association of CTHRC1 with IL-1β and IL-6 in a mouse model of arthritis (25).

**Table 5:** Correlation of blood cell composition and blood analytes in RA patients.

	**CTHRC1**	**Ne**	**Eo**	**Ba**	**MON**	**IL-1β**	**IL-6**	**IL-8**
**Ne**	0.57^*§^							
**Eo**	0.19	0.24						
**Ba**	0.02	0.07	0.05					
**MON**	0.18	0.54^*^	−0.29	0.03				
**IL-1β**	0.88^****^	0.44	0.15	−0.06	0.16			
**IL-6**	0.59^****^	0.24	0.26	−0.15	0.08	0.45^*^		
**IL-8**	0.86^****^	0.37	0.43	0.11	0.13	0.81^****^	0.45^*^	
**IFNγ**	0.91^****^	0.43	0.25	−0.04	0.25	0.86^****^	0.65^**^	0.83^****^

§ *Statistically significant Spearman's correlation rank coefficients **ρ** are labeled in bold. The corresponding p-values for **ρ** coefficients are presented with asterisks as follows*:

* *p < 0.05*,

** *p < 0.01*,

**** *p < 0.0001*.

*Ba, basophil; Eo, eosinophil; IL-1β, interleukin 1 beta; IL-6, interleukin 6, IL-8 interleukin 8, IFNγ, interferon gamma; MON, monocyte; Ne, neutrophil*.

## Discussion

There is an unmet need for specific and easy-to-measure biomarkers to diagnose RA patients and identify patients with high disease activity who are at increased risk of developing erosive, joint destructive disease. In this pilot cross-sectional study, we show that CTHRC1 is a sensitive serological marker for RA that warrants further investigation. We initially identified CTHRC1 through the genetic association of CTHRC1 gene polymorphisms with attenuation of proteoglycan-induced and collagen antibody-induced murine arthritis (24–27). In these mouse models, CTHRC1 plasma levels positively correlated with disease severity and with inflammatory mediators (IL1β and IL-6). In the present study, we extend these earlier findings to human subjects and show that CTHRC1 protein is significantly and specifically elevated in the plasma of RA patients and exhibits positive correlation with RF and ACPA autoantibodies and the acute phase protein CRP. Importantly, CTHRC1 plasma levels were low or undetectable in healthy controls, as well as in OA and ReA patients. Therefore, CTHRC1 may be instrumental as an easy-to-measure plasma marker that can improve RA diagnosis and discriminate RA from OA and potentially other forms of arthritis with an inflammatory component. In patients with RA, plasma levels of CTHRC1 also positively correlated with disease activity. However, because the present study predominantly included patients with moderate disease activity, this association needs to be further investigated and validated through additional studies that include larger groups of patients in remission and patients with high disease activity.

Additional studies will also be required to determine whether and how changes in *Cthrc1* expression are linked to RA pathology and disease progression. The major diagnostic markers and risk factors for severity of joint destruction relate to autoantibodies (anti-CCP/ACPA and RF) or measures of systemic inflammation (CRP, ESR). Several lines of evidence (see below) suggest that CTHRC1 is not simply associated with systemic inflammation, but may be responsive to erosive disease in RA, which is a hallmark of structural damage and is associated with RA disease activity (33). If true, CTHRC1 may represent an independent disease indicator with additive value to the detection of RF, ACPA, and CRP.

Increased expression of CTHRC1 during inflammatory conditions is an uncommon feature of this protein, which is normally expressed during embryonic development (34). In adults, CTHRC1 appears to be expressed mainly in tissues which undergo remodeling including myocardium, the renal arteries, injured skin, differentiated smooth muscle, as well as in osteoblasts, osteoclasts and osteocytes (31, 35, 36), which likely explains the low circulating levels of the protein in healthy controls (37). In mouse models of arthritis, CTHRC1 protein is detected at high levels at the site of joint or bone destruction and may be secreted from activated synoviocytes of the arthritic pannus (28). Based on our preliminary studies, the pannus itself may also be one source for the high CTHRC1 plasma levels observed in RA patients, as elevated CTHRC1 levels were detected in synovial fluid of RA patients and in isolated, cadherin 11-positive (CDH11^+^) RA-FLS (unpublished observation). It is currently unclear whether elevated *Cthrc1* expression reflects a protective role in rheumatic joint disease or a role in disease pathogenesis. In support of the latter, recent studies on the RA synovium revealed specific expression of *Cthrc1* in two different FLS subpopulations, one of which, characterized by the lack of CD34 and the presence of THY1 and CDH11 (CD34^−^ THY1^+^ CDH11^+^), is invasive, significantly expanded in RA vs. OA (38), and promotes osteoclastogenesis, which is a key contributer to RA pathogenesis by deregulating normal bone homeostasis in favor of bone resorption (33). Thus, CTHRC1 may be a marker for invasive FLS associated with disease progression. However, CTHRC1 is also secreted from osteoclasts and modulates the crosstalk between osteoblasts and osteoclasts to couple bone resorption to formation (31, 36, 39–42). Consequently, loss of CTHRC1 function in mice leads to decreased bone mass, decreased osteoblast number and reduced bone formation due to impaired coupling processes, whereas transgenic mice overexpressing *Cthrc1* display high bone mass due to enhanced osteoblastic bone formation (39, 41, 43). In a collagen antibody-induced arthritis model, CTHRC1 was anti-inflammatory and inhibited osteoclast differentiation, as well as joint destruction (41) indicating that CTHRC1 may be part of a protective repair mechanism activated in the inflamed synovium in response to joint and bone erosion.

Our study has several limitations, including a small sample size, particularly in healthy control and ReA patient groups, as well as the homogeneity of treatment and relatively narrow spectrum of disease activity within our RA cohort. The vast majority of the RA population in our study was under treatment with methotrexate derivatives alone or in combination with other therapies. This treatment regimen might limit the potential use of CTHRC1 as a marker for RA. Therefore, inclusion of treatment-naive patients in future studies will be critical as will be the determination of CTHRC1 plasma levels in patients treated with different therapeutics including biological DMARDs. An outstanding question is also the nature of patients with RA diagnosis, but negative for CTHRC1. Studies with expanded cohorts and larger panels of markers will be needed to determine whether CTHRC1 is a marker for a specific subpopulation of RA patients. Moreover, owing to its cross-sectional design, it is difficult to define the relationship between CTHRC1 and RA disease onset and progression. A longitudinal study will be required to assess *Cthrc1* expression in response to disease progression, activity and treatment, and to confirm its potential link to synovitis and bone destruction.

## Conclusion

Here, we identify CTHRC1 as a novel candidate biomarker for RA. At the current level of understanding, CTHRC1 may be instrumental as an easy-to-measure plasma marker that can significantly improve the diagnosis of RA and distinguish RA from OA and other forms of arthritis with an inflammatory component. Our results validate CTHRC1 for future studies focusing on its potential as a marker for RA, as well as its physiological role in bone/cartilage erosion.

## Ethics Statement

The study protocol was approved by the Institutional Research Ethics review board at the Republican Diagnostic Center in Astana, Kazakhstan (granted to Dr. RA, Head of the Department of Internal Medicine and Surgery), and the Institutional Research Ethics Committee at Nazarbayev University, Astana, Kazakhstan (Protocols #N32 and 47, granted to Drs. VA and JK). The study complied with the International Ethical Guidelines for Biomedical Research Involving Human Subjects, Good Clinical Practice guidelines, the Declaration of Helsinki, and local laws. All patients provided written informed consent for the study in accordance with the Declaration of Helsinki.

## Consent for Publication

All authors read and approved the final version for submission and publication.

## Author Contributions

VA, AM, and RA designed the study, contributed to sample collection and data analysis. RA and BY performed clinical work and collected biological samples. AM, YB, AA, and BM helped with sample collection, processing, repository and database organization. AM, YB, AA, ZA, RA, VA, and JK performed data analysis and interpretation. JK was involved in drafting the article or critically revising it. JK had full access to all of the data in the study and takes responsibility for the integrity of the data and the accuracy of the data analysis.

### Conflict of Interest Statement

The authors declare that the research was conducted in the absence of any commercial or financial relationships that could be construed as a potential conflict of interest.

## Abbreviations

ACPA: anti-citrullinated protein antibodies
AUC: area under the curve
BA: basophils
CDH11: Cadherin 11
CD34: Hematopoietic progenitor cell antigen CD34
CI: confidence interval
CRP: C-reactive protein
CTHRC1: Collagen triple helix repeat containing 1
DAS28: disease activity score
DMARDs: Disease-modifying anti-rheumatic drugs
EO: eosinophils
ESR: erythrocyte sedimentation rate
FLS: fibroblast-like synoviocytes
IFN-γ: interferon gamma
IL-1β: interleukin 1 beta
IL-6: interleukin 6
IL-8: interleukin 8
IQR: interquartile range
MBDA: Multi-Biomarker Disease Activity
MON: monocytes
MTX: methotrexate
GC: glucocorticoids
NE: neutrophils
NPV: negative predictive value
NSAIDs: non-steroidal anti-inflammatory drugs
OR: odds ratio
PBS: phosphate-buffered saline
PBS-TB: PBS containing 0.05% Tween 20, 0.1% BSA
PLT: platelets
PPV: positive predictive value
RF: rheumatoid factor
rhCTHRC1: recombinant human CTHRC1
ROC: receiver operating characteristic
S.E.: standard error
SF: synovial fluid
SDS: sodium dodecyl sulfate
SDS-PAGE: sodium dodecyl sulfate polyacrylamide gel electrophoresis
SSZ: sulfasalazine
TMB: 3,3',5,5'- tetramethylbenzidine chromogenic substrate
THY1: thymus cell antigen 1
WNT: Wingless/Integrated.
