# Supplementary material for: CTHRC1: A New Candidate Biomarker for Improved Rheumatoid Arthritis Diagnosis
Source: Front Immunol. 2019 Jun 12;10:1353. doi: 10.3389/fimmu.2019.01353 (PMC6582781; doi:10.3389/fimmu.2019.01353)
Supplement: Supplementary file 1 [file Table_1.DOCX]

| **Patient** | **Gender** | **Age** | **DAS28-CRP** | **Plasma CTHRC1 (ng/ml)** | **Synovial fluid CTHRC1 (ng/ml)** |
| --- | --- | --- | --- | --- | --- |
| RA-1 | F | 69 | 4.59 | 14.3 | 17.5 |
| RA-2 | F | 26 | 4.82 | 21.9 | 15.7 |
| RA-4 | F | 66 | 4.4 | 83.0 | 71.7 |
| RA-7 | F | 44 | 3.86 | 35.2 | 153.7 |
| RA-9 | F | 59 | 5 | 81.4 | 6.3 |

**Table 1S. CTHRC1 levels in plasma and synovial fluid of RA patients. N=5.**
