# Supplementary material for: CTHRC1: A New Candidate Biomarker for Improved Rheumatoid Arthritis Diagnosis
Source: Front Immunol. 2019 Jun 12;10:1353. doi: 10.3389/fimmu.2019.01353 (PMC6582781; doi:10.3389/fimmu.2019.01353)
Supplement: Supplementary file 2 [file Table_2.DOCX]

**Table 2S. Spearman rank correlation coefficents and p-values; N=57**

| ***ρ***  **_______**  ***P*** | **Age** | **WBC** | **HGB** | **RBC** | **PLT** | **Ba** | **LYMF** | **MON** | **Ne** | **Eo** | **ESR** | **CRP** | **RF** | **ACPA** | **DAS28** | **CTHRC1** |
| --- | --- | --- | --- | --- | --- | --- | --- | --- | --- | --- | --- | --- | --- | --- | --- | --- |
| **Age** |  | -0.027 | 0.056 | -0.330 | -0.296 | 0.022 | -0.017 | 0.031 | -0.025 | 0.272 | 0.128 | -0.105 | -0.055 | 0.157 | -0.009 | -0.012 |
|  |  | 0.842 | 0.677 | 0.012 | 0.025 | 0.872 | 0.903 | 0.822 | 0.856 | 0.041 | 0.343 | 0.437 | 0.686 | 0.245 | 0.945 | 0.928 |
| **WBC** | -0.027 |  | 0.054 | 0.307 | 0.384 | 0.445 | 0.534 | 0.588 | 0.795 | 0.252 | -0.024 | 0.455 | -0.014 | 0.088 | 0.308 | 0.200 |
|  | 0.842 |  | 0.692 | 0.020 | 0.003 | 0.001 | 0.000 | 0.000 | 0.000 | 0.059 | 0.858 | 0.000 | 0.920 | 0.516 | 0.020 | 0.137 |
| **HGB** | 0.056 | 0.054 |  | 0.683 | -0.300 | 0.126 | 0.041 | -0.005 | 0.044 | -0.073 | -0.381 | -0.318 | -0.061 | -0.013 | -0.247 | 0.103 |
|  | 0.677 | 0.692 |  | 0.000 | 0.023 | 0.352 | 0.763 | 0.969 | 0.745 | 0.587 | 0.004 | 0.016 | 0.652 | 0.925 | 0.064 | 0.447 |
| **RBC** | -0.330 | 0.307 | 0.683 |  | 0.137 | 0.347 | 0.278 | 0.164 | 0.351 | -0.013 | -0.401 | 0.021 | -0.060 | 0.041 | -0.041 | 0.218 |
|  | 0.012 | 0.020 | 0.000 |  | 0.308 | 0.008 | 0.036 | 0.222 | 0.007 | 0.922 | 0.002 | 0.877 | 0.659 | 0.762 | 0.762 | 0.104 |
| **PLT** | -0.296 | 0.384 | -0.300 | 0.137 |  | 0.230 | 0.148 | 0.189 | 0.384 | 0.165 | 0.136 | 0.507 | 0.090 | 0.030 | 0.292 | 0.134 |
|  | 0.025 | 0.003 | 0.023 | 0.308 |  | 0.085 | 0.273 | 0.160 | 0.003 | 0.220 | 0.312 | 0.000 | 0.508 | 0.822 | 0.027 | 0.321 |
| **Ba** | 0.022 | 0.445 | 0.126 | 0.347 | 0.230 |  | 0.404 | 0.428 | 0.449 | 0.398 | -0.129 | 0.244 | -0.103 | 0.135 | 0.267 | 0.035 |
|  | 0.872 | 0.001 | 0.352 | 0.008 | 0.085 |  | 0.002 | 0.001 | 0.000 | 0.002 | 0.338 | 0.067 | 0.444 | 0.315 | 0.045 | 0.795 |
| **LYMF** | -0.017 | 0.534 | 0.041 | 0.278 | 0.148 | 0.404 |  | 0.559 | 0.421 | 0.178 | -0.070 | 0.224 | -0.124 | 0.036 | 0.140 | -0.059 |
|  | 0.903 | 0.000 | 0.763 | 0.036 | 0.273 | 0.002 |  | 0.000 | 0.001 | 0.185 | 0.606 | 0.093 | 0.358 | 0.789 | 0.299 | 0.664 |
| **MON** | 0.031 | 0.588 | -0.005 | 0.164 | 0.189 | 0.428 | 0.559 |  | 0.589 | 0.228 | -0.117 | 0.413 | 0.105 | 0.172 | 0.317 | 0.169 |
|  | 0.822 | 0.000 | 0.969 | 0.222 | 0.160 | 0.001 | 0.000 |  | 0.000 | 0.089 | 0.384 | 0.001 | 0.437 | 0.202 | 0.016 | 0.209 |
| **Ne** | -0.025 | 0.795 | 0.044 | 0.351 | 0.384 | 0.449 | 0.421 | 0.589 |  | 0.337 | 0.045 | 0.471 | 0.013 | 0.073 | 0.347 | 0.235 |
|  | 0.856 | 0.000 | 0.745 | 0.007 | 0.003 | 0.000 | 0.001 | 0.000 |  | 0.010 | 0.739 | 0.000 | 0.925 | 0.590 | 0.008 | 0.078 |
| **Eo** | 0.272 | 0.252 | -0.073 | -0.013 | 0.165 | 0.398 | 0.178 | 0.228 | 0.337 |  | -0.120 | 0.105 | -0.255 | 0.248 | 0.014 | -0.021 |
|  | 0.041 | 0.059 | 0.587 | 0.922 | 0.220 | 0.002 | 0.185 | 0.089 | 0.010 |  | 0.373 | 0.437 | 0.056 | 0.063 | 0.917 | 0.875 |
| **ESR** | 0.128 | -0.024 | -0.381 | -0.401 | 0.136 | -0.129 | -0.070 | -0.117 | 0.045 | -0.120 |  | 0.283 | 0.106 | -0.226 | 0.409 | -0.088 |
|  | 0.343 | 0.858 | 0.004 | 0.002 | 0.312 | 0.338 | 0.606 | 0.384 | 0.739 | 0.373 |  | 0.033 | 0.432 | 0.091 | 0.002 | 0.515 |
| **CRP** | -0.105 | 0.455 | -0.318 | 0.021 | 0.507 | 0.244 | 0.224 | 0.413 | 0.471 | 0.105 | 0.283 |  | 0.204 | 0.130 | 0.789 | 0.305 |
|  | 0.437 | 0.000 | 0.016 | 0.877 | 0.000 | 0.067 | 0.093 | 0.001 | 0.000 | 0.437 | 0.033 |  | 0.128 | 0.335 | 0.000 | 0.021 |
| **RF** | -0.055 | -0.014 | -0.061 | -0.060 | 0.090 | -0.103 | -0.124 | 0.105 | 0.013 | -0.255 | 0.106 | 0.204 |  | 0.437 | 0.358 | 0.596 |
|  | 0.686 | 0.920 | 0.652 | 0.659 | 0.508 | 0.444 | 0.358 | 0.437 | 0.925 | 0.056 | 0.432 | 0.128 |  | 0.001 | 0.006 | 0.000 |
| **ACPA** | 0.157 | 0.088 | -0.013 | 0.041 | 0.030 | 0.135 | 0.036 | 0.172 | 0.073 | 0.248 | -0.226 | 0.130 | 0.437 |  | 0.155 | 0.350 |
|  | 0.245 | 0.516 | 0.925 | 0.762 | 0.822 | 0.315 | 0.789 | 0.202 | 0.590 | 0.063 | 0.091 | 0.335 | 0.001 |  | 0.249 | 0.008 |
| **DAS28** | -0.009 | 0.308 | -0.247 | -0.041 | 0.292 | 0.267 | 0.140 | 0.317 | 0.347 | 0.014 | 0.409 | 0.789 | 0.358 | 0.155 |  | 0.312 |
|  | 0.945 | 0.020 | 0.064 | 0.762 | 0.027 | 0.045 | 0.299 | 0.016 | 0.008 | 0.917 | 0.002 | 0.000 | 0.006 | 0.249 |  | 0.018 |
| **CTHRC1** | -0.012 | 0.200 | 0.103 | 0.218 | 0.134 | 0.035 | -0.059 | 0.169 | 0.235 | -0.021 | -0.088 | **0.305^§^** | **0.596^§^** | **0.350^§^** | **0.312^§^** |  |
|  | 0.928 | 0.137 | 0.447 | 0.104 | 0.321 | 0.795 | 0.664 | 0.209 | 0.078 | 0.875 | 0.515 | **0.021** | **<0.0001** | **0.008** | **0.018** |  |

Spearman's rank correlation analysis was used to analyze relationships between changes in individual clinical parameters and plasma CTHRC1 levels. Spearman's rank correlation coefficient *ρ* is shown for each individual measure (dark blue shaded boxes). The corresponding *P*-value (*P*) is presented below (light blue shaded boxes). p<0.05 is considered as statistically significant.

^§^ Statistically significant Spearman’s correlation coefficients *ρ* and p-values for CTHRC1 are labeled in a boldface font.

**ESR**, erythrocyte sedimentation rate; **CRP**, C-reactive protein; **RF**, rheumatoid factor; **ACPA**, anti-citrullinated protein antibodies; **DAS28-CRP**, disease activity score based on CPR; **WBC**, white blood cells; **HGB**, hemoglobin; **RBC**, red blood cells; **PLT**, platelet; **Ba**, basophil; **LYMF**, lymphocyte; **MON**, monocyte; **Ne**, neutrophil; **Eo**, eosinophil.
